# Effect of an education programme for patients with osteoarthritis in primary care - a randomized controlled trial

**DOI:** 10.1186/1471-2474-11-244

**Published:** 2010-10-25

**Authors:** Eva Ekvall Hansson, Malin Jönsson-Lundgren, Anne-Marie Ronnheden, Eva Sörensson, Åsa Bjärnung, Leif E Dahlberg

**Affiliations:** 1 Lund University, Department of Clinical Sciences/Family Medicine, Malmö University Hospital, Malmö, Sweden; 2 Primary Health Care in Malmö, Rehabilitation Unit, Abels Rehab, Malmö, Sweden; 3 Lund University, Department of Clinical Sciences/Orthopaedics, Malmö University Hospital, Malmö, Sweden

## Abstract

**Background:**

Osteoarthritis (OA) is a degenerative disease, considered to be one of the major public health problems. Research suggests that patient education is feasible and valuable for achieving improvements in quality of life, in function, well-being and improved coping. Since 1994, Primary Health Care in Malmö has used a patient education programme directed towards OA. The aim of this study was to evaluate the effects of this education programme for patients with OA in primary health care in terms of self-efficacy, function and self-perceived health.

**Method:**

The study was a single-blind, randomized controlled trial (RCT) in which the EuroQol-5D and Arthritis self-efficacy scale were used to measure self-perceived health and self-efficacy and function was measured with Grip Ability Test for the upper extremity and five different functional tests for the lower extremity.

**Results:**

We found differences between the intervention group and the control group, comparing the results at baseline and after 6 months in EuroQol-5D (p < 0.001) and in standing one leg eyes closed (p = 0.02) in favour of the intervention group. No other differences between the groups were found.

**Conclusion:**

This study has shown that patient education for patients with osteoarthritis is feasible in a primary health care setting and can improve self-perceived health as well as function in some degree, but not self-efficacy. Further research to investigate the effect of exercise performance on function, as well as self-efficacy is warranted.

**Trial registration:**

The trial is registered with ClinicalTrials.gov. Registration number: NCT00979914

## Background

Osteoarthritis (OA) is a degenerative disease, considered to be one of the major public health problems [1]. The predominant symptoms are pain, stiffness and impaired quality of life along with psychological distress [2]. Treatment often consists of medication. Later in the disease, joint replacement surgery commonly occurs. Cartilage as well as function and quality of life can be influenced positively by physical exercises [3-5]. Physical exercise may also reduce the need for hospital care after knee joint replacement [6].

Patient education programmes are often defined as a planned learning experience to influence a patient's knowledge and health behaviour [7]. Education can be given by a physician as part of a consultation, in small groups or delivered by multidisciplinary team [7,8]. Research suggests that patient education is feasible and valuable in terms of improvements in quality of life, function, well-being and improved coping [9-13]. In research on patient education for OA, several different forms of patient education can be implemented, some of them only self-management programmes, some only exercise programmes and some programmes combining self-management and exercise. Systematic reviews have been performed which address the different types of programmes, with varying conclusions [9,14,15]. Devos-Comby et al concluded that self-management programmes had little effect on function but somewhat more on psychological health [9]. Walsh et al concluded that programmes with a combination of exercises and self-management programmes and exercises combined could reduce pain and increase function [15]. In contrast, Chodosh et al concluded that self-management programmes for OA did not have any effect on pain or function [14]. One difference in the systematic reviews is the addition of exercise to traditional self-management, which might explain the contradictory results. Despite the uncertainty in findings, guidelines recommend education as a core treatment for osteoarthritis [16].

Since 1994, Primary Health Care in Malmö has used a patient education programme directed towards OA and delivered by a multi-disciplinary team. The programme has been developed by physiotherapists and occupational therapists in primary health and includes information on exercise and self-management.

The aim of this study was to evaluate the effects of this education programme for patients with OA in primary health care in terms of self-efficacy, function and self-perceived health.

## Methods

### Patients

The patients were referred to the patient education programme for osteoarthritis (PEPOA) by their GP, orthopaedic specialist, physiotherapist or occupational therapist. Inclusion criteria in the study were patients of any age with OA in the knee, hip or hand with pain, stiffness and limitation of movement in the affected joint. Exclusion criteria were inability to speak and understand Swedish.

### Outcome measures

The main outcome measure for self-perceived health was the generic instrument EuroQol-5D (EQ5D) [17,18]. EQ5D comprises five dimensions: mobility, self-care, usual activities, pain/discomfort and anxiety/depression. Each dimension has three levels: no problems, some problems, severe problems. The patient indicates his/her state by placing a cross in the box for the most appropriate statement. This result in a 1-digit number and the digits for the five dimensions can be combined in a five-digit number, generating 243 possible combinations of responses. The EQ5D can be presented as a health profile or as a global health index with a weighted total value (British tariff used), where the minimum value is -0.594 and the maximum 1.0 [19]. The EQ5D also consists of a Visual Analogue Scale (EQ5D-VAS), where patients are asked to rate their health on a vertical scale, where 100 is best imaginable health state, and 0 is worst imaginable health state. EQ5D is widely used for monitoring health status among different patient groups all over the world [18]. It has been used for measuring outcome of intervention among patients with OA [20], with chronic conditions [21] and as a predictor of outcome after hip replacement surgery [22].

The main outcome measure for self-efficacy was the generic instrument Arthritis Self-Efficacy Scale (ASES) [23]. ASES consists of three items: pain, function and other symptoms. For each item there are a number of questions about how confident the patient feels about performing an activity or a task. Answers are given on a visual analogue scale, where 10 is not confident at all and 100 very confident. The mean value for each item is calculated. ASES has been used for evaluating patient education programmes for patients with arthritis [23] and for patients with chronic pain, and the Swedish version has been tested for reliability and validity [24,25].

As the main outcome measure for function in the lower extremities, one-leg rising from sitting to standing was used, with the patient being asked to rise from a stool, 48 cm high, as many times as possible. If the patient was not able to rise from 48 cm, one-legged rising from 60 cm was used. Bipedal rising from sitting to standing and one-legged jump were used as secondary outcome measures for function in the lower extremities. In bipedal rising from sitting to standing, the patient was asked to rise from a stool, 48 cm high, using both legs, as many times as possible. Bipedal rising has been used for assessing patients with fibromyalgia syndrome [26]. In one-legged jump, [27] the distance from heel in the starting position to heel in the landing position was measured. The one-legged jump has been used for assessing patients after meniscectomy [28]. To measure balance performance, standing one leg eyes open (SOLEO) and standing one leg eyes closed (SOLEC) were used. In SOLEO and SOLEC the patient was standing with arms crossed [29]. The time, up to 60 seconds was measured. SOLEO and SOLEC have been used in primary care settings [30,31]. Bipedal rising, single-leg standing, SOLEO and one-legged jump have been tested for reliability [26,28,32]. Bipedal rising has shown acceptable interrater reliability (mean 1.1, SD 2.0, correlation coefficients 0.96, interrater coefficient variation 7.6) [26], SOLEO has shown high interrater reliability (Spearman Rank-Order Correlations -0.38 to -0.45) [32] and one-legged jump has shown good test-retest reliability (ICC 0.92, 95%CI 0.86 to 0.96 and 0.93, 95% CI 0.87 to 0.97) [28].

The main outcome measure for function in the upper extremity was the Grip Ability Test (GAT) [33]. GAT consists of three items; putting a flexigrip stocking over the non-dominant hand, putting a paper clip on an envelope and pouring water from a jug. The time for each item is measured and calculated in a total score. A high score corresponds to decrease in hand function. GAT has been tested for reliability and internal consistency (intraobserver test r = 0.985, p < 0.001; interobserver test r = 0.948, p < 0.001; internal consistency (Cronbach's alpha) 0.65) [33] and has been used for patients with rheumatoid arthritis [34].

All measures were performed in the same order for each patient, both at baseline and at follow-up.

### Randomization process

All patients were tested at baseline with the aforementioned clinical measures and completed ASES and EQ-5D. Tests at baseline were performed by either a physiotherapist or an occupational therapist. After completion of the assessment, the tester informed the patient about the study. If the patient agreed to participate, informed consent was obtained. Then an independent person randomized the patient to either the intervention group or the control group, using a random number list and sealed envelopes.

### Intervention

The intervention group participated in PEPOA and the control group continued living as usual. PEPOA consists of five group sessions, three hours for each session, once a week for five weeks. The focus was on self-efficacy. Eight to ten patients participated in the programme at the same time. The programme is described in detail in table 1.

**Table 1:** The patient education programme for osteoarthritis (PEPOA)

Session	Content
First session	Physiotherapist and occupational therapist at the same time. Information about anatomy, about physiology of pain and coping with pain. Try cold and heat. Brainstorming about what the participants finds hard to do.
Second session	Physiotherapist. Information about exercise and physical activity. Practical demonstration of home-training exercises for the lower extremity. Demonstration of different kinds of orthopaedic aids for the lower extremity.
Third session	Orthopaedic specialist, nurse and nutritionist. Information about OA and current research. Information about medications. Information about appropriate diet.
Fourth session	Occupational therapist. Ergonomics and practical instructions about equipment and technical aids. Feedback to the brainstorming from session one.
Fifth session	Occupational therapist. Information about surgery of the hand, demonstration of orthopaedic aids for hands. Try out treatment with hot paraffin. Practical demonstration of home training exercises for the hand.

The programme lasted for five weeks, with group sessions once a week, three hours for each session.

After six months, all patients were assessed with the same tests as at baseline, as well as ASES and EQ-5D. An independent person performed the six-month tests. After these measures, the patients in the control group were invited to participate in PEPOA.

In the planning of the present study, a pilot study was performed where we found the intervention as well as the measures suitable for a larger study [35].

## Statistics

Considering a standard deviation of 12 for one-leg rising [27], 13.3 for SOLEO, 7.4 for SOLEC [36], 0.18 for EQ5D [37] and the assumed clinically relevant difference of 3 for SOLEC [31], 0.09 for EQ5D [37] and one standard deviation for the other tests, a power of 80% at significance level 0.05, it was deemed that a sample size of 100 persons was required [38].

Since data were normally distributed, the one-way ANOVA was used to calculate differences between the two groups when data were quantitative. The Wilcoxon signed ranked test was used for calculating differences between the two groups in the proportion of levels in EQ5D. Since a visual analogue scale is a construct scale, the Wilcoxon signed ranked test was also used to calculate differences between the groups [39]. Data were calculated with an intention-to-treat analysis. The significance level was set at p ≤ 0.05.

The data package SPSS version 16.0 was used (SPSS Inc, software location Lund University).

## Ethics

The study was approved by the Regional Ethical Review Board in Lund.

## Results

A total of 120 patients were assessed for eligibility in the study, of whom 114 patients agreed to participate and were randomized to either intervention group or control group. Six patients did not want to participate in the study and were excluded before randomization. A total of 100 patients were assessed after 6 months.

**Figure 1:**
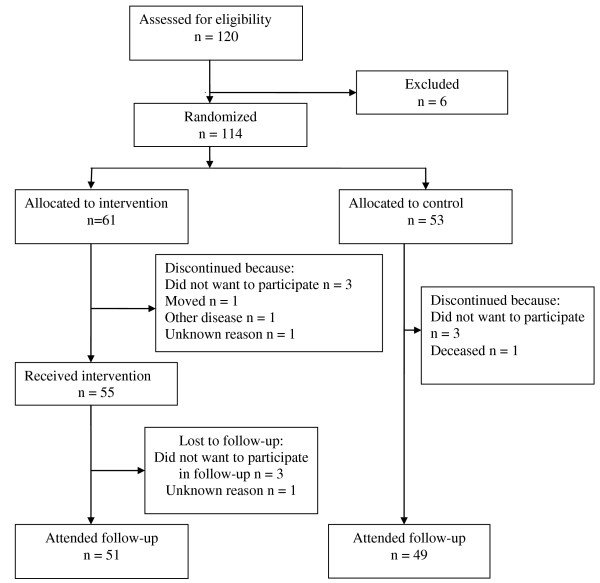
**Flowchart of the study**.

The 114 patients (97 women) who were included in the study were between 41 and 84 years old, mean age 63. The most common OA-affected joints were knee and hand. Sixty-eight per cent of the patients had BMI 25 or more. Baseline data for intervention group and control group are shown in table 2. As seen in table 2, there were no statistically significant differences between the groups at baseline. Fourteen patients did not complete the study, 10 in the intervention group and 4 in the control group. The patients who dropped out were between 47 and 81 years old, mean 63 years. There were 13 women and one man in the dropout group. A flow-chart of the study is shown in figure 1.

**Table 2:** Baseline data for the study group, mean values, range and standard deviation (SD).

	Intervention			Control			
		
	n = 61			n = 53			
**Measures**	**Mean**	**Range**	**SD**	**Mean**	**Range**	**SD**	**p**

BMI	28.31	18.59-46.72	5.42	27.82	20.61-43.58	5.00	0.41
Age	62	42-81	9.43	63	41-84	9.51	0.83
ASES-Pain	57.47	10-98	20.49	54.33	10-100	23.62	0.33
ASES-Function	74.81	34-100	17.37	73.59	28-100	19.88	0.35
ASES-Other symtoms	66.78	15-100	19.43	67.31	17-100	23.68	0.12
EQ5D-index	0.58	-0.022-0.79	0.25	0.56	-0.13-1.01	0.30	0.14
EQ5D-VAS	63.52	0-100	21.81	65.73	20-100	22.19	0.62
GAT total	22.87	12-79.39	10.09	24.67	14-47.81	7.83	0.45
GAT 1	7.89	1.81-39.59	5.52	7.91	3.62-18.01	3.29	0.47
GAT 2	4.28	2.00-14.02	2.48	5.12	2.03-16.19	2.68	0.18
GAT 3	10.49	5.38-28.82	3.72	11.90	5.41-28.79	4.58	0.19
SOLEO (sec)	33.55	0-60	22.02	35.71	0-60	23.00	0.60
SOLEC (sec)	6.18	0-30	6.31	6.82	0-30	7.09	0.58
One-legged jump (cm)	34.78	0-147	33.29	33.11	0-98	29.90	0.63
One-legged raising (as many times as possible)	9.00	0-31	8.89	12.03	0-48	12.73	0.83
Bipedal raising (as many times as possible)	15.00	3-50	12.10	13.10	0-30	8.40	0.61
OA location* (number)	3/21/21/16			2/18/16/17			0.71
Smokers (number)	5			9			0.36
BMI groups 20-25/25-30/>30 (number)	22/24/16			18/20/15			0.41

* hip/knee/hand/more than one location.

Results in function, self-efficacy and self-perceived health after 6 months are shown in table 3 and table 4. There were statistically significant differences between the two groups in EQ5D levels in the five dimensions, with the intervention group having a higher proportion of patients in level 1 (no problems) after 6 months in all the dimensions (mobility, self-care, usual activities, pain/discomfort and anxiety/depression) (p < 0.001). There were also statistically significant differences between the groups in EQ5D-VAS (p = 0.02), where the intervention group had improved more than the control group, but there was no statistically significant difference in ASES (table 3 and table 4).

**Table 3:** Mean changes from baseline, mean difference, 95% CI of difference, p-value.

	Intervention n = 61	Control n = 53			
				
	mean change	mean change	diff	(CI)	p
GAT total (points)	-1.52	-1.69	0.17	(-2.56 to 2.91)	0.90
GAT 1 (points)	-0.88	-0.06	0.82	(-2.43 to 0.79)	0.32
GAT 2 (points)	-0.61	-1.24	0.63	(-0.21 to 1.46)	0.14
GAT 3 (points)	0.10	-0.53	0.63	(-0.69 to 2.07)	0.31
SOLEO (sec)	-1.35	-3.94	2.59	(-3.29 to 8.47)	0.38
SOLEC (sec)	0.57	-1.13	1.17	(0.33 to 3.06)	**0.02**
One-legged jump (cm)	-4.07	-7.55	3.48	(-7.08 to 14.04)	0.51
One-legged rising (times)	2.33	-1.08	3.41	(-2.07 to 8.89)	0.22
Bipedal rising (times)	1.30	-3.91	5.19	(-0.53 to 10.92)	0.10
ASES-Pain	4.94	4.08	0.86	(-6.72 to 8.44)	0.82
ASES-Function	3.89	-0.39	4.25	(-1.42 to 10.07)	0.14
ASES-Other symptoms	4.85	0.92	3.93	(-2.41 to 10.27)	0.23
EQ5D-index	0.07	0.00	0.07	(-0.02 to 10.17)	0.17
EQ5D-VAS	5.59	1.18	3.73	(-3.01 to 10.47)	**0.02**

In GAT, decrease means improvement, in all other tests, increase means improvement.

**Table 4:** Proportion of levels 1, 2 and 3 in EQ5D by dimension and group at baseline and at test after 6 months.

EQ5D dimensions		Intervention baseline/6 months %	Control baseline/6 months %	p
Mobility	Level 1*	46/54	47/47	**<0.001**
	Level 2	54/46	53/53	
	Level 3	0/0	0/0	
Self-care	Level 1	90/89	89/81	**<0.001**
	Level 2	10/11	9/17	
	Level 3	0/0	2/2	
Usual activities	Level 1	57/76	60/58	**<0.001**
	Level 2	41/22	34/40	
	Level 3	2/0	6/2	
Pain/discomfort	Level 1	0/3	5/6	**<0.001**
	Level 2	84/84	78/73	
	Level 3	16/13	17/21	
Anxiety/depression	Level 1	40/57	37/41	**<0.001**
	Level 2	60/43	53/50	
	Level 3	0/0	10/9	

P-values for the difference in change between baseline and 6 months, between the two groups.

*Level 1 indicates no problems, level 2 indicates some problems and level 3 indicates extreme problems.

There were statistically significant differences between intervention group and the control group after 6 months in SOLEC (p = 0.02), where the intervention group had improved more than the control group (table 3).

## Discussion

### Main findings

In this randomized controlled trial, we found improvements in SOLEC (p = 0.02), in EQ5D-VAS (p = 0.02) and in proportions of patients who were at the different levels in EQ5D (p < 0.001) after a patient education programme for osteoarthritis.

### Limitations of the study's methods

As in all randomized controlled trials concerning rehabilitation, it is not possible to use a double-blind design, since the patients always know whether they are in the intervention group or the control group. The present study has a single-blind design, with measurements at baseline and after six months performed by either a physiotherapist or an occupational therapist, who did not know whether the patient had been in the intervention group or the control group. This design possibly minimizes the risk of confounding factors. Also, to make sure that the assessor was blinded, another assessor performed tests after 6 months. Since the outcome measures have been tested for reliability, we do not think that this has compromised the results [26,28,32]. However, being thoroughly assessed is a form of intervention that may make the patient aware of his or her weaknesses, such as poor ability to rise from a chair. Therefore, it is possible that the patients in the control group performed exercises by themselves, or found information about osteoarthritis on their own.

Measures were performed at baseline and after 6 months. After baseline measures, there was a waiting time for participation in PEPOA of approximately one to three months. This means that the six-month measures were performed between one and a half and three and a half months post-treatment for the intervention group. It is possible that this has diluted our findings.

### Limitations of the study's results

The information about height and weight was given by the patients. Since weight is one of the factors that people tend to underestimate, it is possible that this is the case in our study too. However, still more than one third of the patients were overweight and one third was obese.

GAT was designed for patients with rheumatoid arthritis and is perhaps not sensitive enough to measure differences in function among patients with osteoarthritis, which might be considered in future studies. Also, our patients come from a primary care setting, and even if 60% of the patients had hand OA, their baseline measure in GAT was better than in patients with rheumatoid arthritis, after one year of anti-TNF therapy [40]. It is possible that our patients reached the ceiling for GAT already at baseline.

In EQ5D, changes from level 3 to level 2 give more index weight than changes from level 2 to level 1. In our study, the proportion of patients at level 3 in the 5 different dimensions at baseline was low. This may explain the lack of statistically significant differences in change in EQ5D index, even though more patients improved from level 2 to level 1 in the intervention group. Also, EQ5D has shown to be less sensitive to change than other QOL measures [41] which also might explain the lack of significant results in our study.

In the intervention, the first session of the PEPOA contained a brainstorming about what the participants found was hard to do in daily life. In the fourth session, there was feedback on the brainstorming. The intention was that these components in the PEPOA should affect self-efficacy in a positive way, but no improvements in self-efficacy were seen. Thus, brainstorming and feedback on brainstorming seems to be insufficient for affecting self-efficacy and another approach than used in our PEPOA is probably needed.

There were two options of one-leg rising, either from 48 cm or from 60 cm. There were 87 patients who managed to rise from 48 cm, 47 in the intervention group and 40 in the control group. Since the same height was used for measures after 6 months as at baseline, we do not think that this has influenced our results.

Patient education programmes have been shown to improve self-efficacy and coping skills among patients with low back pain [42], but not among patients with arthritis in primary health care [43]. Our programme did not include exercises, but we still found significant differences in function and in self-perceived health, although not in self-efficacy. Earlier reviews have led to contradictory conclusions about the effect of patient education programmes for OA on function [9,14,15]. A patient education programme for patients with knee pain has been shown to reduce the number of consultations with general practitioners [44], a variable that we have not considered in our study. It seems that our programme succeeded in improving self-perceived health but not self-efficacy, and it had only minor influence on function. Nevertheless, it seems feasible in primary health care.

### Dropouts

There were a total of 14 dropouts in the study. Data were analysed on an intention-to-treat basis, with the dropouts included and the last observation carried forward [45]. There were more dropouts in the intervention group than in the control group, 10 in the intervention group and 4 in the control group. In the intervention group, 6 patients dropped out before receiving intervention and 4 after receiving intervention. Reasons for dropping out were similar in the intervention group and in the control group. Therefore we do not think that the intervention itself affected whether the patient dropped out or not.

## Conclusion

This randomized controlled trial has shown that patient education for patients with osteoarthritis is feasible in a primary health care setting and can improve self-perceived health as well as function in some degree, but not self-efficacy. The current programme included education rather than instruction in exercise, and further research to investigate the effect of exercise performance on function, as well as self-efficacy is warranted.

## Competing interests

The authors declare that they have no competing interests.

## Authors' contributions

EEH participated in the design of the study, performed the statistical analysis and drafted the manuscript. MJL, AMR, ES and ÅB participated in data collection. LED participated in the design of the study and helped to draft the manuscript. All authors read and approved the final manuscript, except for ES, who very sadly has passed away.

## Pre-publication history

The pre-publication history for this paper can be accessed here:

http://www.biomedcentral.com/1471-2474/11/244/prepub
